# An Adaptive Sparse Subspace Clustering for Cell Type Identification

**DOI:** 10.3389/fgene.2020.00407

**Published:** 2020-04-28

**Authors:** Ruiqing Zheng, Zhenlan Liang, Xiang Chen, Yu Tian, Chen Cao, Min Li

**Affiliations:** ^1^School of Computer Science and Engineering, Central South University, Changsha, China; ^2^Departments of Biochemistry & Molecular Biology and Medical Genetics, Alberta Children's Hospital Research Institute, University of Calgary, Calgary, AB, Canada

**Keywords:** single cell RNA-seq, subspace clustering, adaptive sparse strategy, similarity learning, visualization

## Abstract

The rapid development of single-cell transcriptome sequencing technology has provided us with a cell-level perspective to study biological problems. Identification of cell types is one of the fundamental issues in computational analysis of single-cell data. Due to the large amount of noise from single-cell technologies and high dimension of expression profiles, traditional clustering methods are not so applicable to solve it. To address the problem, we have designed an adaptive sparse subspace clustering method, called AdaptiveSSC, to identify cell types. AdaptiveSSC is based on the assumption that the expression of cells with the same type lies in the same subspace; one cell can be expressed as a linear combination of the other cells. Moreover, it uses a data-driven adaptive sparse constraint to construct the similarity matrix. The comparison results of 10 scRNA-seq datasets show that AdaptiveSSC outperforms original subspace clustering and other state-of-art methods in most cases. Moreover, the learned similarity matrix can also be integrated with a modified t-SNE to obtain an improved visualization result.

## 1. Introduction

Cells are the basic functional unit all organisms are made of and play significant roles in the different stages of life. Through various DNA and RNA sequencing data, researchers have a comprehensive and deep understanding of cell biology. However, traditional sequencing data is obtained from bulks of cells, and these are composed of the mixed effect of numerous cells and ignore cell heterogeneity. These bulk-seq data will lead to deviations in downstream analysis if a specific type of cell is expected. Recently, single-cell sequencing techniques have developed rapidly and make up the defect of bulk sequencing data. Although the single-cell sequencing technique cannot capture all cell information, it provides a great opportunity to reveal the characteristics of an individual cell.

The fundamental step of analyzing the single-cell data is to identify the cell types. Utilizing single-cell RNA-seq (scRNA-seq) data to obtain the cell clusters is one of the most efficient methods available. The amount of clustering methods on the basis of scRNA-seq data have been proposed. A group of methods are focused on calculating more accurate and robust similarity scores between cells. SNN-cliq (Xu and Su, 2015) constructed the distance matrix and counted the number of common neighbor cells for each pair of cells as the similarity scores and then incorporated these within a clique-based clustering method. Seurat (V3.0) was inspired by an SNN-cliq and applied the SNN graph with a louvain algorithm (Butler et al., 2018; Stuart et al., 2019). Seurat is one of the most widely used methods. SIMLR (Wang et al., 2017) and SC3 (Kiselev et al., 2017) adopted multiple similarity metrics from different aspects. In SIMILR, we could learn the inherent similarity matrix from a different resolution of Gaussian kernels, while SC3 combined multiple sub-clustering results together to build up a consensus matrix. Random forest (Pouyan and Kostka, 2018) was another way to calculate the similarity. The correlation coefficient has been proven to be effective when estimating the pairwise similarity of cells, and a high-order correlation coefficient was also applied in the scRNA-seq data analysis (Jiang et al., 2018; Tang et al., 2019). Compared to the methods based on pair-wise distance or correlation measurement, SinNLRR (Zheng et al., 2019b) considered the subspace characteristics of cells' expression and assumed the low rank and non-negative properties of the similarity matrix. Besides, several methods, including nonnegative matrix factorization (NMF) (Shao and Höfer, 2017; Zhu et al., 2017), imputation, and dimensionality reduction-based methods (Yau et al., 2016; Lin et al., 2017), have been used widely in assessing cellular heterogeneity. In the other aspect, the increasing number of well-learned scRNA-seq datasets also drives the appearance of supervised methods. These methods depended on labeled training datasets or some prior biological knowledge, such as gene markers (Wagner and Yanai, 2018; Pliner et al., 2019). According to the latest study (Abdelaal et al., 2019), most of the supervised methods are sensitive to prior knowledge, dataset complexity, or input features. Moreover, this kind of method has a fixed resolution and cannot find the detailed subtypes from a rough cell group. In this study, we have focused on the unsupervised clustering methods to identify the cell types. Inspired by previous methods, calculating the distance or similarity matrix of cells is a critical step. To recognize more accurate similarities of cells from high dimensional expression profiles, we have proposed an adaptive sparse subspace clustering method called AdaptiveSSC. AdaptiveSSC follows the subspace assumption and remains the nearest neighbors of a cell by a data-driven adaptive sparse constraint. The derived similarity matrix is used to obtain the clustering result and visualization. AdaptiveSSC obtains an improved performance on multiple experimental datasets.

## 2. Materials and Methods

**Figure 1:**
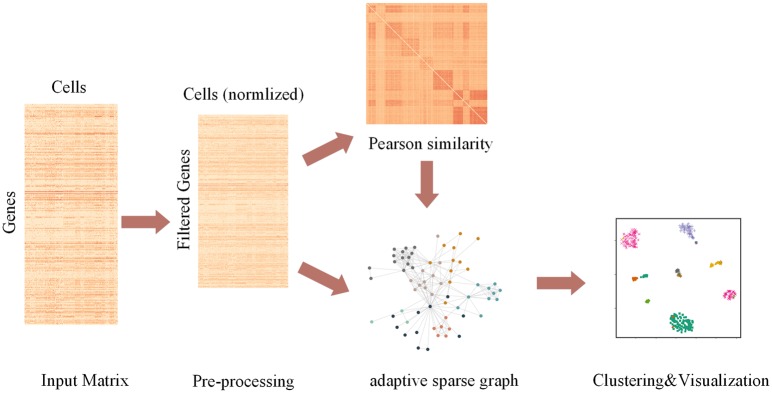
The pipeline of AdaptiveSSC to identify and visualize cell types from scRNA-seq data.

The pipeline of AdaptiveSSC is shown in Figure 1. Taking the scRNA-seq expression matrix as the input, AdaptiveSSC constructs the sparse cell-to-cell similarity matrix by keeping the most similar cells for each cell before then applying it to spectral clustering and modified t-distributed stochastic neighbor embedding (t-SNE) to obtain cell groups and the visualization result.

### 2.1. Data Pre-processing

The quantified scRNA-seq data contain thousands of genes, and the sparsity of gene expression is usually high. Therefore, AdaptiveSSC filters the genes expressed in <10% of the cells (the maximum number is 100), which are not regarded as informative genes. AdaptiveSSC investigates the linear effect of other cells on the target cell. To remove the scale of cells' expression, the *L*_2_ normalization is carried on the original gene expression matrix.

(1) $$\begin{array}{l} X_{ij}=G_{ij}/\sqrt{\overset{M}{\underset{k=1}{\sum }}G_{kj}^{2}} \end{array}$$

where *G* is the original expression matrix with *M* genes and *N* cells. The normalized matrix *X* is used in the following calculation.

### 2.2. Adaptive Sparse Subspace Clustering

Most clustering methods depend on the calculation of the similarity or distance matrix. The most popular similarity measurements include Euclidean distance, Pearson or Spearman correlations, and cosine similarity, which are all based on a pairwise estimation. The scRNA-seq data usually contains thousands of genes; however, only a part of a gene determines the cell type, which corresponds to a low-dimensional manifold surface. According to the common strategy in manifold learning, only the local measurement of similarity or distance is reliable, so previous scRNA-seq clustering methods (Xu and Su, 2015; Wang et al., 2017) usually apply k-nearest neighbors (KNN) to keep the locality. However, the KNN is used arbitrary to select the same number of neighbors for each cell, and the selection of *k* would have a great influence on the final result in some situations. In order to overcome these shortcomings, we propose an adaptive sparse subspace clustering method, which we have called AdaptiveSSC.

AdaptiveSSC is developed from sparse subspace clustering (SSC) methods. SSC is proposed to solve the motion segmentation and face clustering problems (Elhamifar and Vidal, 2013). SSC assumes that the feature vector of a sample can be expressed as the linear combination of other samples in the same subspace or type. Based on the assumption, the expression of a cell *X*_*i*_ = *c*_1_*X*_1_ + *c*_2_*X*_2_ + ⋯+*c*_*i*−1_*X*_*i*−1_ + *c*_*i*+1_*X*_*i*+1_ + ⋯+*c*_*N*_*X*_*N*_ and *c*_*k*_ is the subspace coefficient denoting the similarity score between cells. If the cell *i* and *k* are the same type, *c*_*k*_ > 0, otherwise it is 0. By adding *l*_1_ term, the most similar cells lying in the same subspace are retained. Extending it to all cells, the calculation of the subspace coefficient matrix is defined as Equation (2):

(2) $$\begin{array}{l} min{\left|C\right|}_{1}\;s.t,\;X=XC\text{ and }diag\left(C\right)=0 \end{array}$$

where *X* is the normalized expression matrix. *C* is the coefficient matrix and *C*_*ij*_ denotes similarity between cell *i* and *j*. |·|_1_ denotes *l*_1_ norm. The larger values in *C* mean the more similar cells. The relaxation formula of the optimization problem is shown:

(3) $$\begin{array}{l} min\frac{1}{2}\|X-XC{\|}_{F}^{2}+\lambda {\left|J\right|}_{1}\;s.t,\;diag\left(C\right)=0\text{ and }C-J=0 \end{array}$$

where $||\cdot |{|}_{F}^{2}$ means the Fresenius norm and λ is the *l*_1_ penalty factor, which controls the sparsity of the coefficient matrix. *J* is an auxiliary matrix.

In the Equation (3), the coefficient matrix *C* is sensitive to the selection of the *l*_1_ penalty factor. Another problem is that the same penalty factor for all coefficients will lead to the loss of consistency between estimation and variable selection (Zou, 2006). Therefore, we have introduced a data-driven adaptive strategy to solve these problems. As a Pearson correlation has been proven to be effective when measuring the similarity in previous studies (Kiselev et al., 2017; Wang et al., 2017), we utilized it to adjust the penalty factor for each coefficient. If the correlation of two cells is high, the penalty factor is decreased and vice versa. The modified optimization problem is therefore defined:

(4) $$\begin{array}{l} \begin{array}{l} min\frac{1}{2}\|X-XC{\|}_{F}^{2}+\text{λ}|\frac{J}{W}{|}_{1}\;s.t,\;diag(C)=0\text{ and }C-J=0 \end{array}\\ \text{ where},\;W_{ij}=\{\begin{array}{ll} pearson\left(X_{i},X_{j}\right)\text{If} & pearson\left(X_{i},X_{j}\right)>0\\ 0 & \text{otherwise} \end{array} \end{array}$$

where $\frac{J}{W}$ means element division of matrix *J* and *W*. We set the negative value of the Pearson correlation to 0. Because only the trend of the expression of two cells are positively correlated, we regard them as similar cells. Some zero values in *W* would lead to zero values in *J* during the optimization.

Alternating direction method of multipliers (ADMM) (Boyd et al., 2011) is an efficient method to solve Equation (4). According to ADMM, the augmented Lagrangian formula is defined:

(5) $$\begin{array}{l} \iota_{\gamma ,\lambda }\left(C,J,Y\right)=\frac{1}{2}{\left\|X-XC\right\|}_{F}^{2}+\lambda |\frac{J}{W}{|}_{1}+tr\left(Y^{T}\left(C-J\right)\right)\\ \;+\frac{1}{2\gamma }{\left\|C-J\right\|}^{2}\text{ and }diag\left(C\right)=0 \end{array}$$

where *Y* is a dual variable, γ is an augmented Lagrangian penalty parameter, and *tr* means the trace of the matrix. ADMM updates *C*, *Y*, or *J* by fixing others. In iteration *k* + 1, the optimized form of *C*^*k*+1^, *J*^*k*+1^, and *Y*^*k*+1^ is shown in Equations (6–8):

(6) $$\begin{array}{l} \begin{array}{l} C^{k+1}={\left(X^{T}X+\frac{1}{\gamma }I\right)}^{-1}\left(X^{T}X+\frac{1}{\gamma }\left(J^{k}-Y^{k}\right)\right)\\ C^{k+1}=C^{k+1}-diag\left(C^{k+1}\right) \end{array} \end{array}$$

(7) $$\begin{array}{l} \begin{array}{l} J^{k+1}=threshold_{\frac{\lambda }{W},\gamma }\left(C^{k+1}+Y^{k}\right)\\ \;=sign\left(C^{k+1}+Y^{k}\right)\cdot max\left(|C^{k+1}+Y^{k}|-\frac{\lambda }{\gamma W},0\right)\\ J^{k+1}=J^{k+1}-diag\left(J^{k+1}\right) \end{array} \end{array}$$

(8) $$\begin{array}{l} \begin{array}{c} Y^{k+1}=Y^{k}+\frac{1}{\gamma }\left(C^{k+1}-J^{k+1}\right) \end{array} \end{array}$$

where *sign*() means the sign function. The convergence of ADMM mainly includes primal residuals and dual residuals. On the basis of updating process, the penalty parameter γ affects the speed of convergence. In AdaptiveSSC, we apply a balance strategy (Boyd et al., 2011) between primal residuals and dual residuals to adjust γ. The setting of γ is shown:

(9) $$\gamma_{k+1}=\{\begin{array}{lll} \gamma_{k}/2, & \text{when} & {\left\|r^{k}\right\|}_{2}>\mu {\left\|s^{k}\right\|}_{2},\\ \;2\gamma_{k}, & \text{when} & {\left\|s^{k}\right\|}_{2}>\mu {\left\|r^{k}\right\|}_{2},\\ \;\gamma_{k}, & \text{others}. & \; \end{array}$$

where *r*^*k*^ = *C*^*k*^ − *J*^*k*^ is the primal residual and $s^{k}=\frac{1}{\gamma }\left(J^{k}-J^{k-1}\right)$ is the dual residual. The μ is set to 50 as default. To reduce the computational complexity, γ is updated by 10 iterations. When *max*(*abs*(*C* − *J*)) < 0.0001 or the number of iteration is larger than 200, this update process is finished. To keep the symmetry of the similarity matrix, the final similarity matrix *S* = *C*^*T*^ + *C*.

Finally, the spectral clustering (SC) (Von Luxburg, 2007) is applied on the learned similarity matrix. The SC is based on the point of graph cut and utilizes the characteristic of the corresponding Laplacian matrix to divide the graph into several clusters. In AdaptiveSSC, we use the normalized Laplacian matrix $L^{norm}=I-D^{-\frac{1}{2}}SD^{-\frac{1}{2}}$, where *D* is the degree matrix, to obtain its *k* eigenvectors corresponding to the smallest *k* eigenvalues. Then, k-means is used to obtain the final clusters.

## 3. Results and Discussion

### 3.1. scRNA-seq Datasets

We collected 10 scRNA-seq datasets to evaluate the performance of AdaptiveSSC. These datasets are based on different single-cell techniques or protocols, such as Smart-seq, SMARTer, and Drop-seq based methods. Meanwhile, the scale of these datasets ranges from the tens to the tens of thousands. The variety of the datasets could indicate the generalization ability of AdaptiveSSC comprehensively. The details of these datasets are shown in Table 1. All datasets contain the real cell types from the original researches.

**Table 1:** Single cell RNA-seq datasets.

**Datasets**	**Cell number**	**Gene number**	**Techniques**
Darmanis (Darmanis et al., 2015)	420	22,085	SMARTer
Kolod (Kolodziejczyk et al., 2015)	704	10,685	Smart-Seq2
Treutlein (Treutlein et al., 2014)	80	959	SMARTer
Yan (Yan et al., 2013)	90	20,214	Tang et al., 2011
Ting (Ting et al., 2014)	114	14,405	Single CTC RNA-Seq
Engel (Engel et al., 2016)	203	23,337	Smart-seq2
Kumar (Kumar et al., 2014)	361	11,497	SMARTer
Vento (Vento-Tormo et al., 2018)	5,418	33,693	Smart-seq2
Baron (Baron et al., 2016)	8,569	20,125	inDrop
Shekhar (Shekhar et al., 2016)	26,830	13,166	Drop-seq

### 3.2. Evaluation Metrics

In order to compare the performance of different clustering methods, we selected two popular metrics: normalized mutual information (NMI) and adjusted rand index (ARI). Both NMI and ARI can quantify the consistency between the clustering results and the real labels. The definition of NMI and ARI is shown:

(10) $$\begin{array}{l} NMI\left(T,P\right)=\frac{I\left(T,P\right)}{\left[H\left(T\right)+H\left(P\right)\right]} \end{array}$$

(11) $$ARI\left(T,P\right)=\frac{\sum_{ij}\left(\begin{array}{c} n_{ij}\\ 2 \end{array}\right)-\left[\sum_{i}\left(\begin{array}{c} n_{i.}\\ 2 \end{array}\right)\sum_{j}\left(\begin{array}{c} n_{.j}\\ 2 \end{array}\right)\right]/\left(\begin{array}{c} n\\ 2 \end{array}\right)}{\frac{1}{2}\left[\sum_{i}\left(\begin{array}{c} n_{i.}\\ 2 \end{array}\right)+\sum_{j}\left(\begin{array}{c} n_{i.}\\ 2 \end{array}\right)\right]-\left[\sum_{i}\left(\begin{array}{c} n_{i.}\\ 2 \end{array}\right)\sum_{j}\left(\begin{array}{c} n_{i.}\\ 2 \end{array}\right)\right]/\left(\begin{array}{c} n\\ 2 \end{array}\right)}$$

Where *T* and *P* mean the real labels and clustering labels, respectively. In Equation (11), *n*_*ij*_ denotes the number of cells belonging to *i* group in real labels and *j* group in clustering labels; *n*_*i*_ denotes the number of cells belonging to the *i* group in real labels, while *n*_*j*_ denotes the number of cells belonging to the *j* group in clustering labels.

### 3.3. Parameter Analysis

**Figure 2:**
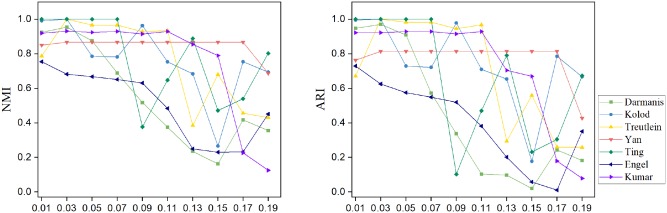
The corresponding NMI and ARI with different values of λ on eight datasets.

Although the adaptive strategy is used in AdaptiveSSC, there are still some hyperparameters to be set. The most important hyperparameter is the *l*_1_ penalty factor λ. By the adaptive adjustment, the learned similarity matrix is not so sensitive to it. We evaluated the NMI and ARI of AdaptiveSSC on eight small datasets (smaller than 5,000 cells) with λ ranging from 0.01 to 0.19 and the interval set to 0.02. The results for eight small datasets are shown in Figure 2. Based on the result, when the λ was in the 0.01–0.05, both NMI and ARI were in the best range and were more stable. Therefore, we used λ = 0.03 as a default in AdaptiveSSC. During the experiment, we also found the optimal λ was not consistent for big datasets (in Baron is 0.01 and in Shekhar and Vento is 0.007). We recommend that users select the proper λ by grid searching with the following rule. If the corresponding sparsity of *C* is between 0.02 and 0.05, the λ should be selected. In Baron and Shekhar, we selected the corresponding λ with the sparsity of *C* is 0.03.

### 3.4. Comparison Analysis of Clustering Methods

**Figure 3:**
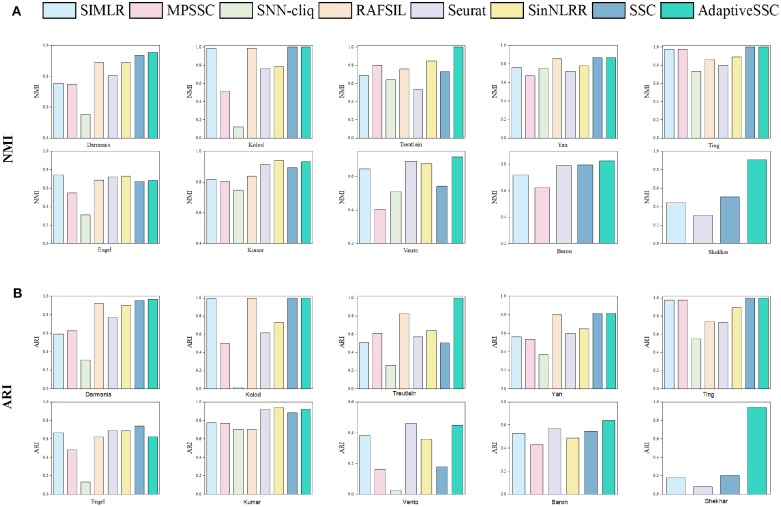
The corresponding **(A)** NMI and **(B)** ARI of SIMLR, MPSSC, SNN-cliq, RAFSIL, Seurat, SinNLRR, SSC, and AdaptiveSSC on 10 datasets.

To validate the effectiveness of AdaptiveSSC, we selected seven competitive methods, including SIMLR (Wang et al., 2017), MPSSC (Park and Zhao, 2018), SNN-cliq (Xu and Su, 2015), RAFSIL (Pouyan and Kostka, 2018), Seurat(V3.0) (Butler et al., 2018; Stuart et al., 2019), SinNLRR (Zheng et al., 2019b), and sparse subspace clustering (SSC) (Elhamifar and Vidal, 2013). All these methods are based on the construction of similarity matrix. SNN-cliq and Seurat recalculate the similarities based on their shared neighbors. SIMILR and MPSSC focus on the different resolution of Gaussian kernels, while RAFSIL applies random forest. SinNLRR is based on the subspace assumption with low rank constraint. The original SSC was selected as the baseline method. The results of NMI and ARI on 10 datasets are shown in Figure 3. Compared to SSC, AdaptiveSSC improved NMI and ARI in six datasets. Especially in Treutelin, Kumar, Vento, and Shekhar, AdaptiveSSC exhibited a significant improvement, more so than SSC, which means the adaptive penalty factor leads to the more accurate similarity matrix. In Kolod and Ting, AdaptiveSSC achieved the same performance with SSC. Overall, AdaptiveSCC exhibited a better performance than SSC in most cases. Besides, AdaptiveSSC achieved the best (or a tie for first place) performance in seven datasets upon NMI and eight datasets upon ARI compared with other six state-of-the-art methods. It is worth noting that only AdaptiveSSC obtains the perfect result on Treutelin. The results in Baron and Shekhar also verify AdaptiveSSC's effectiveness in large datasets. Estimation of the number of cell types is another important aspect in application. In AdaptiveSSC, we also used *eigengap* to determine the number of clusters, which was popular in previous studies. The results can be found in the Supplementary Material. As shown in the results, none of the methods predict the correct number of clusters in all datasets. However, AdaptiveSSC obtains the correct number of clusters in three datasets and gets the closest number in five datasets, which is a better selection overall. Moreover, we select five different scale datasets to evaluate the computational efficiency of these methods. The running time can be found in the Supplementary Material. AdaptiveSSC has a faster speed than SSC but is still time-consuming in large datasets compared with SIMLR and Seurat. All the experiments run on the server with 24 cores and 512 GB memory. The methods with running time more than 36 h are excluded, such as RAFSIL, SNN-cliq, and SinNLRR in large scale datasets, and MPSSC gets out of memory error on Shekhar.

### 3.5. Comparison Analysis of Visualization

**Figure 4:**
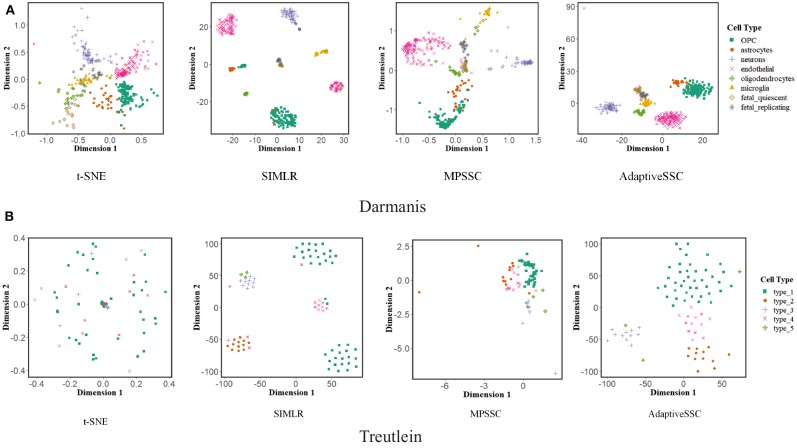
The visualization of t-SNE, SIMLR, MPSSC, and AdaptiveSSC on **(A)** Darmanis and **(B)** Treutelin.

Visualization of scRNA-seq is another important issue. Previous study (Wang et al., 2017) proposed a modified t-distributed stochastic neighbor embedding (t-SNE) to validate the performance of learned similarity. We also adopted this evaluation to AdaptiveSSC and generate 2D-embedding images on Darmanis and Treutelin with the learned similarity matrix of t-SNE, SIMLR, MPSSC, and AdaptiveSSC, respectively. The result is shown in Figure 4. The points with the same color mean they have the same cell type. Compared to other methods, AdaptiveSSC could group the same cells together and exhibits good silhouettes. Although SIMILR and MPSSC contain more dense parts, they divide cells with same type into different cliques, which are usually far away from each other. This will give the researchers a misconception that they are belong to exactly different types. Therefore, AdaptiveSSC has a better performance and potential in the visualization of scRNA-seq data.

### 3.6. Discussion and Conclusion

The identification of cell types is a fundamental problem is scRNA-seq data analysis. In recent years, a lot of clustering methods have been proposed to solve it. However, most of these methods do not exhibit a good generalization on different datasets. In this study, we proposed a subspace clustering with an adaptive sparse constraint, called AdaptiveSSC. AdaptiveSSC regards the expression of a cell can be expressed as a linear combination of other cell's expression from the same type. A data-driven adaptive sparse strategy is applied to keep the locality of cells in the original dimension and decrease the sensitivity to the penalty factor. Eight scRNA-seq datasets were used to evaluate the performance of AdaptiveSSC. By comparing with SSC, AdaptiveSSC improves the clustering results significantly in some cases, which indicates the effectiveness of our strategy. Moreover, six state-of-the-art methods were selected as comparison. From the NMI and ARI, AdaptiveSSC achieves the best performance in most of datasets. Finally, we integrated the learned similarity with modified t-SNE further, which also shows the powerful potential of AdaptiveSSC in visualization.

However, the computational efficiency of AdaptiveSSC is still low for large datasets and should be improved in the future. Some strategies used in the fast clustering method could be considered to make AdaptiveSSC more efficient (Ren et al., 2019). Moreover, AdaptiveSSC explores the cell heterogeneity from a gene level, but it is also important to study the different biological functions of cells. Regulatory modules (Aibar et al., 2017) have been proved effective when showing the functional heterogeneity of cells. It is possible to identify the cell type from the whole gene regulatory network perspective (Li et al., 2017; Zheng et al., 2018, 2019a). Besides, motivated by previous studies (Lan et al., 2018; Chen et al., 2019; Shi et al., 2019), multi-view learning and integrating with prior knowledge are promising directions to improve the accuracy of clustering and give a higher resolution of cell types.

## Data Availability Statement

Publicly available datasets were analyzed in this study. This data can be found here: https://github.com/zrq0123/AdaptiveSSC.

## Author Contributions

RZ and CC designed the methodology. RZ, ZL, XC, and YT run the comparison experiments on datasets. RZ and ML wrote the paper. All authors revised and approved the manuscript.

## Conflict of Interest

The authors declare that the research was conducted in the absence of any commercial or financial relationships that could be construed as a potential conflict of interest.
