# Supplementary material for: An Adaptive Sparse Subspace Clustering for Cell Type Identification
Source: Front Genet. 2020 Apr 28;11:407. doi: 10.3389/fgene.2020.00407 (PMC7212354; doi:10.3389/fgene.2020.00407)
Supplement: Supplementary file 1 [file Data_Sheet_1.PDF]

Supplementary Material

1 SUPPLEMENTARY TABLES

1.1 Comparison analysis of estimating the number of clusters

We compare the predicted number of clusters in these datasets. In SIMLR, MPSSC, RAFSIL, SinNLRR, SSC and AdaptiveSSC, the *eigengap* method are used to predict the number of clusters based on the learned similarity matrix, and SNN-Cliq and Seurat determine the number by their own methods. As shown in the results, none of the methods predicts the correct number of clusters in all datasets. However, AdaptiveSSC obtains the correct number of clusters in three datasets and gets the closest number in five datasets, which is the better selection overall.

Table S1. The estimated number of cell types. - means the running time exceeds 36 hours on the sever

Datasets	True number	SIMLR	MPSSC	SNN-Cliq	RAFSIL	Seurat	SinNLRR	SSC	AdaptiveSSC
Darmanis	8	13	18	17	1	8	9	8	8
Kolod	3	9	1	3	1	7	5	5	6
Treutlein	5	10	15	14	1	2	3	4	4
Yan	6	12	16	18	1	3	7	16	5
Ting	5	5	15	8	1	4	4	5	5
Engel	4	3	2	13	1	4	4	1	1
Kumar	4	3	3	8	2	4	4	3	5
Vento	38	11	1	178	-	18	10	40	30
Baron	14	5	1	-	-	15	-	23	6
Shekhar	5	15	-	-	-	20	-	1	5

1.2 Comparison analysis of computational time

We compare the running time of eight methods on the same sever. According to the results, the running time of AdaptiveSSC requires lower than original SSC, which means the balance strategy is effective. In small datasets (Yan, Darmanis and Kolod), AdaptiveSSC is faster than other methods. However, in large datasets (Baron and Shekhar), SIMLR and Seurat is much more efficient than AdaptiveSSC. For 20K cells (Shekhar), AdaptiveSSC needs more than 4 hours. The computational efficiency of AdaptiveSSC needs the further improvement in large datasets.

Table S2. The comparison of computational time for eight methods (in seconds). - means the time exceeds 36 hours on the sever

Datasets	SIMLR	MPSSC	SNN-Cliq	RAFSIL	Seurat	SinNLRR	SSC	AdaptiveSSC
Yan	1.48	2.99	1.38	6.11	20.87	1.32	2.73	0.96
Darmanis	10.18	4.38	7.07	154.09	22.318	14.1548	36.67	4.28
Kolod	24.65	10.48	385.09	195.33	17.88	32.51	22.43	6.84
Baron	48.32	3269.2	-	-	134.25	20967.05	2955.88	815.49
Shekhar	195.28	-	-	-	129.87	-	32344.21	15607.58
